# Measurement of Shear Strain Field in a Soft Material Using a Sensor System Consisting of Distributed Piezoelectric Polymer Film

**DOI:** 10.3390/s20123484

**Published:** 2020-06-19

**Authors:** Fengyu Li, Yasuhiro Akiyama, Xianglong Wan, Shogo Okamoto, Yoji Yamada

**Affiliations:** Department of Mechanical Systems Engineering, Graduate School of Engineering, Nagoya University, Nagoya 464-8603, Japan; akiyama-yasuhiro@mech.nagoya-u.ac.jp (Y.A.); huochedaxian@yahoo.co.jp (X.W.); okamoto-shogo@mech.nagoya-u.ac.jp (S.O.); yamada-yoji@mech.nagoya-u.ac.jp (Y.Y.)

**Keywords:** shear strain, soft material, embedded sensor, piezoelectric sensor

## Abstract

Measurement of the internal stress and strain distributions within soft materials is necessary in the field of skin contact safety. However, conventional interactive force sensors cannot efficiently obtain or estimate these distributions. Herein, a shear strain sensor system consisting of distributed built-in piezoelectric polyvinylidene fluoride (PVDF) polymer films was developed to measure the internal shear strain field of a soft material. A shear strain sensing model was mathematically established, based on the piezoelectricity and mechanical behavior of a bending cantilever beam, to explain the sensing principle. An experiment in three-dimensional measurement of the shear strain distribution within an artificial skin was designed and conducted to assess the sensitivity of the sensing model. This sensor system could visualize the shear strain field and was sensitive to different contact conditions. The measurement results agreed well with the results of numerical simulation of the substrate, based on contact mechanics. The proposed sensor system was confirmed to provide a new sensing method for the field of shape analysis. The sensor system can be applied to develop sufficiently sensitive electronic skin and can significantly contribute to skin damage analysis and skin contact safety assessment.

## 1. Introduction

Soft materials play important roles in everyday life and in a wide range of technological applications. The mechanical properties of soft materials are characterized in terms of elasticity, plasticity, and viscosity [1]. Some soft materials exhibit anisotropy or multilayer structures. These mechanical properties can make the distributions of stress and strain in soft materials complex. The detection and estimation of the internal conditions of soft materials not only contribute to fundamental science, but also are needed in the field of skin safety assessment [2]. Skin tissue is a soft material that protects against external physical stress and protects the inner vital organs. Excessive loads can cause wounds to skin tissue. This type of mechanical injury has been widely researched [3,4,5], and skin contact safety has also received considerable research attention [6].

With the aging of the global population, the numbers of users of wheelchairs and other mechanical means of assistance, as well as of long-term bedridden patients are increasing yearly. Various friction traumas occur frequently in connection with these situations because of excessive shear stress and friction on skin tissue. In the use of physical assistant robots (PAR), the main power transfer between the human and the robot is through cuffs or belts that are attached firmly to the human body. The long-term repetition of human-robot interactive force that exceeds safe threshold levels generates friction blisters and skin abrasion [7,8,9]. The international safety standard ISO 13482 puts forth a series of safety requirements for personal care robots [10]. Although this safety standard clearly states that degrees of skin–robot friction and shear stress should be reduced to avoid injury, quantitative requirements for achieving this goal are not specifically stated. Pressure ulcers are another type of injury to the skin and underlying tissue that result from prolonged pressure and frictional forces [11]. To clarify the mechanism of skin injury quantitatively and to be able to anticipate skin injury generation, it is important to be able to observe and monitor the stress and strain distributions within soft tissues. Therefore, an effective quantitative measurement technique for the distribution of stress or strain within soft material is needed.

A conventional multi-axis force sensor or accelerometer has the fundamental problem of spatial limitation. Measurements are commonly obtained interactively with such devices and are limited to the interfaces of two contacted or connected parts. For instance, in the PAR skin abrasion tests described by Mao et al., the contact force between the cuff and skin model was obtained using a three-axis force sensor positioned at the cuff-skin interface [12]. Nevertheless, in the field of tactile sensors, various excellent sensing structures have been developed using various sensing techniques [13,14]. Yao et al. proposed a tactile-based approach to determine the center of mass of a target object by applying the OptoForce 3D tactile sensor as the semi-spherical sensitive fingertip based on the optical technology [15]. Kaboli and Feng et al. developed a multi-modal artificial skin containing seven tactile modules to gain more detailed tactile knowledge of new objects based on the proposed active tactile transfer learning algorithm [16,17]. Lenzi et al. applied a matrix of optoelectronic sensors embedded in a thin and compliant silicone mass to a user-robot contact surface to measure the pressure distribution on the interaction area [18]. Noda et al. proposed a tactile sensor with standing piezoresistive cantilevers embedded in an elastic material to detect the shear stress applied to a surface [19]. Moreover, recent research in electronic artificial skin has assembled excellent designs with high sensitivity, stretchability, and reliability [20,21]. Although these measurement targets were still limited in terms of the interactive force, by embedding the sensing element within a substrate, built-in structures as these provide a good starting point for designing a sensor system for internal stress or strain distribution measurements. In this study, we employed a compact and flexible piezoelectric polymer film, polyvinylidene fluoride (PVDF) film, as a built-in sensitive transducer to establish a sensor system for internal stress or strain distribution monitoring.

PVDF is a thin film-type organic polymer with high mechanical flexibility. PVDF-based sensors have been used frequently in industry for vibration monitoring and control and for dynamic pressure sensing [22,23,24]. Yi et al. designed a PVDF-based deformation sensor for use in insect locomotion studies [25]. This type of sensor was later embedded on the inner tread surface of a tire to obtain critical information for understanding and estimating wheel-ground interactions [26]. However, little research has demonstrated PVDF sensors used to monitor the internal stress or strain field of soft material.

In this study, we monitored the internal mechanical conditions of a soft material using a novel sensor system with a distributed built-in sensing element composed of PVDF polymer film. The sensor system was capable of observing the three-dimensional distribution of shear strain within the material directly. The validity of this sensor system was evaluated by comparing the measurement results with those obtained from a mathematical model. Furthermore, the effect of the shape of the contactor on the strain distribution was investigated.

## 2. Soft Material Shear Strain Sensing Methodology

### 2.1. Sensor System Hardware

**Figure 1:**
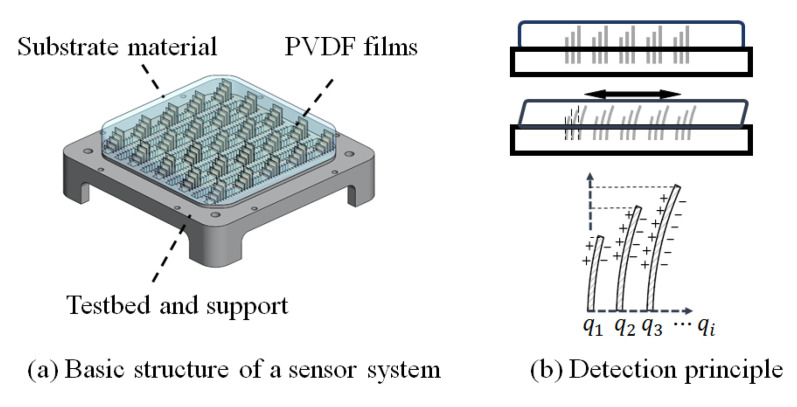
Shear strain measuring device.

The basic structure of the proposed sensor system for shear stress and strain field measurement within a soft material is shown in Figure 1a. The sensing elements of PVDF polymer films were fixed perpendicularly to the surface and embedded in the soft substrate material.

The shear strain sensing methodology used in this study took advantage of the high flexibility and sensitive in-plane sensing mode of PVDF film. When the soft substrate is deformed by an external force, the PVDF films are flexible enough to be deformed along with the substrate. The deformation value is transferred to the charge induced by piezoelectricity, so the actual deformation of the soft substrate can be instantaneously measured by the output piezoelectric charges. To obtain and visualize the three-dimensional internal distribution, therefore, the sensor films should be arranged within the soft substrate with different lengths and different positions, as illustrated in Figure 1b. The feasibility of the measurement method was initially validated by assessing the mechanical characteristic of the soft material [27].

**Figure 2:**
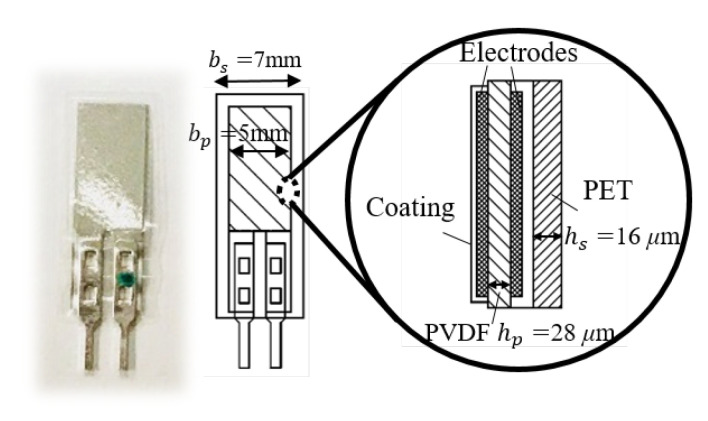
Schematic illustration of the configuration of the PVDF polymer film.

Figure 2 shows a schematic illustration of the configuration of the PVDF polymer film used in this study. The PVDF sensor element consisted of a PVDF film sheet with electrodes on both sides. The electrodes were connected with crimps and solder tabs for affixing wires for signal transmission. Note that the PVDF polymer film was laminated to a thicker package on one side and had a much thinner laminate on the other side. This displaced the neutral axis of the structure from the central axis of the PVDF, which resulted in a tensile strain when deflected downward and a compressive strain when deflected in the opposite direction in the piezo film [28].

### 2.2. Soft Material Shear Strain Sensing Model

The relationship between the output electrical charge from the PVDF polymer films and the soft material shear strain information is mathematically described in this section. A shear strain sensing model was established based on piezoelectricity and the mechanical behavior of a bending cantilever beam. The piezoelectricity effect of the in-plane sensing mode of the PVDF sensor was employed in the sensor system development.

**Figure 3:**
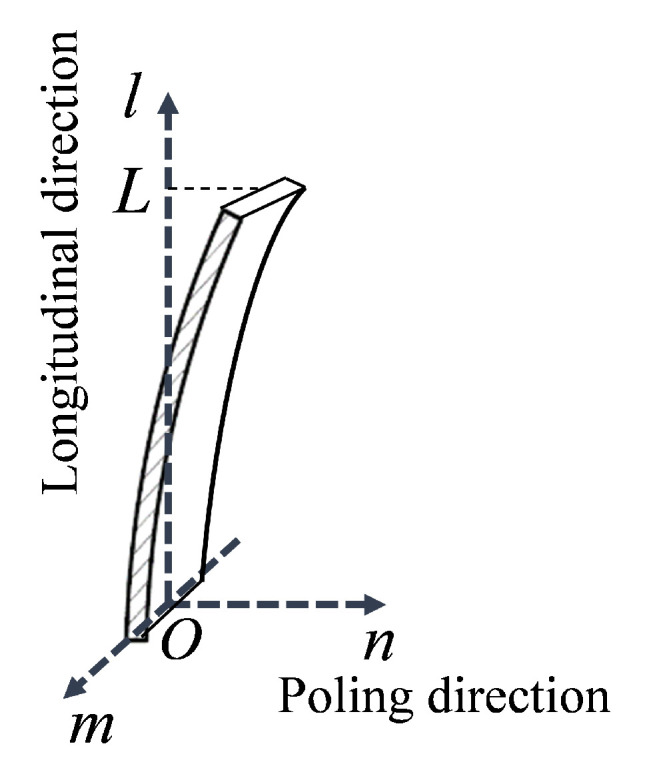
Orientation and geometric deflection of the PVDF polymer film.

**Figure 4:**
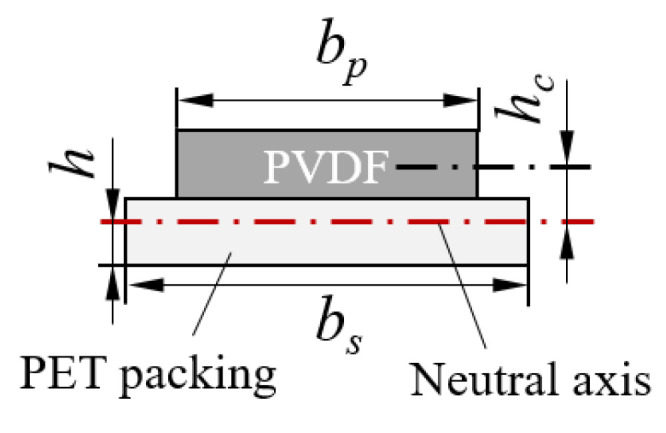
Cross-section of the simplified PVDF structure.

The charge calculation under deflection of the PVDF film was derived from two main constitutive equations, the direct piezoelectricity equation of PVDF [29] and the bending curvature differential equation [30]. According to the constitutive governing equation of piezoelectricity for sheet PVDF, the accumulated electric charge can be calculated as:

(1) $$\begin{array}{c} q=e_{31}\int_{A_{3}}S_{1}\;dA_{3} \end{array}$$

where $e_{31}$ is the piezoelectric coefficient, $S_{1}$ is the bending strain, and $A_{3}$ is the electrode area of the PVDF surface. Subscripts 1 and 3 indicate the longitudinal direction and the poling direction, respectively, responding to the *l* and *n* axes defined in Figure 3. To obtain the expression of the bending strain $S_{1}$ of a cantilever beam from the curvature differential equation, the geometric deflection of the cantilever-based PVDF film in a three-dimensional Cartesian coordinate O−lmn is shown schematically in Figure 3. The origin *O* was chosen at the middle point of the fixed end. The *l* axis lied in the longitudinal direction of the neutral surface in the undeformed configuration. The *m* axis lied in the plane of the structure perpendicular to the *l* axis. The bending strain is expressed in terms of the geometric dimension and deflection as:

(2) $$S_{1}\left(l\right)=-\frac{h_{c}}{\rho (l)}=-h_{c}\frac{n^{\prime \prime }}{{\left[1+{\left(n^{\prime }\right)}^{2}\right]}^{\frac{3}{2}}}$$

where ρ is the curvature radius of the neutral layer, $h_{c}$ is the distance from the center of the PVDF layer to the neutral axis, and *n* is the deflection of the cantilever beam at any section in terms of *l*. $n^{\prime }$ and $n^{\prime \prime }$ represent the first and second derivative of its deflected shape with respect to *l*, respectively. In regards to the position of the neutral layer, which appears in the equations above, the calculation of the distance from the center of the PVDF layer to the neutral axis $h_{c}$ is discussed based on the mechanical structure of the sensor element. The cross-section of the PVDF structure was simplified to a two-layer structure composed of PVDF and a polyester PET packing material, as shown in Figure 4.

The position of the neutral axis is defined as being a distance *h* from the bottom of the PET layer. The widths of the PVDF and the PET sheets are denoted by $b_{p}$ and $b_{s}$, respectively, and the thicknesses of the PVDF and the PET sheets are $h_{p}$ and $h_{s}$, respectively. The specific values of the dimensions can be seen in Figure 2. $E_{p}$ and $E_{s}$ are the corresponding Young’s moduli. The mathematical expression for the equilibrium equations of the moment of the bending beam is written as:

(3) $$E_{p}b_{p}h_{p}\left(\frac{h_{p}}{2}+h_{s}-h\right)+\frac{E_{s}b_{s}{\left(h_{s}-h\right)}^{2}}{2}=\frac{E_{s}b_{s}h^{2}}{2}$$

Because of the similar elastic moduli of the PVDF and PET, $E_{p}≃E_{s}$, the distance from the center of the PVDF layer to the neutral axis can be calculated as:

(4) $$h=\frac{b_{p}{h_{p}}^{2}+b_{s}{h_{s}}^{2}+2b_{p}h_{s}}{2b_{p}h_{p}+2b_{s}h_{s}}$$

(5) $$h_{c}=\frac{h_{p}}{2}+h_{s}-h$$

By substituting $S_{1}$ with Equation (2), the charge generation of Equation (1) can be expressed as:

(6) $$q=-e_{31}\cdot h_{c}\cdot b_{p}\int_{0}^{L}\frac{n^{\prime \prime }}{{\left[1+{\left(n^{\prime }\right)}^{2}\right]}^{\frac{3}{2}}}dl$$

which establishes the relation between the charge and the geometric deflection.

The integral part of Equation (6) is evaluated as follows:

(7) $$\begin{array}{cc} q & =-e_{31}\cdot h_{c}\cdot b_{p}\int_{n^{\prime }\left(0\right)}^{n^{\prime }\left(L\right)}\frac{1}{{\left[1+{\left(n^{\prime }\right)}^{2}\right]}^{\frac{3}{2}}}dn^{\prime }\\ \; & =-e_{31}\cdot h_{c}\cdot b_{p}\frac{n^{\prime }}{\sqrt{1+{\left(n^{\prime }\right)}^{2}}}\left|\overset{n^{\prime }\left(L\right)}{\underset{n^{\prime }\left(0\right)}{}}\right. \end{array}$$

As the deflection at the fixed position is zero, the output charge produced by the PVDF films only corresponds to the gradient of the tip of the free end of the film $n^{\prime }\left(L\right)$ as follows:

(8) $$q=-e_{31}\cdot h_{c}\cdot b_{p}\frac{n^{\prime }\left(L\right)}{\sqrt{1+{\left[n^{\prime }\left(L\right)\right]}^{2}}}$$

Therefore, $n^{\prime }\left(L\right)$ can be expressed as:

(9) $$n^{\prime }\left(L\right)=\frac{q}{\sqrt{{\left(e_{31}h_{c}b_{p}\right)}^{2}-q^{2}}}$$

Based on the sensor system’s properties and assuming that the deflection n(l) of the PVDF film coincided with the internal deformation of the soft substrate, $n^{\prime }\left(L\right)$ rightly represented the shear strain of the substrate at the tip of the free end of the PVDF film. The derived relation explained why the detecting target was shear strain, but not shear stress, which might be initially expected. Therefore, the series of mathematical equations used to describe the shear strain measurement principle using PVDF could explain the application of this sensor system to shear strain monitoring.

### 2.3. Signal Conditioning: Charge Amplifier Circuit

**Figure 5:**
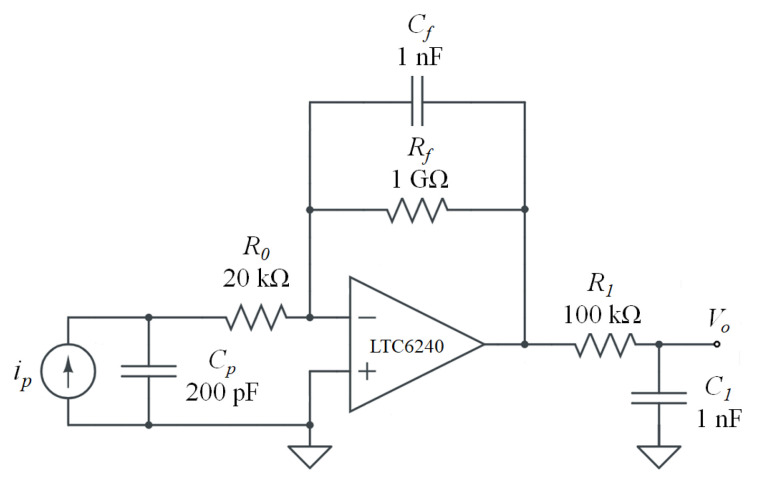
Overall signal conditioning circuit for the PVDF sensor.

As a piezoelectric sensor, the PVDF film is a differential sensor device and can be modeled as an equivalent circuit of a charge generator in parallel with a capacitance. Only one type of PVDF polymer film with a capacitance of 200 pF was applied in this study as shown in the illustration in Figure 2. A feasible charge amplifier circuit was designed and is schematically illustrated by the circuit diagram shown in Figure 5. The circuit was composed of an equivalent circuit of a piezoelectric sensor followed by a charge amplifier and a low-pass filter. The charge amplifier signal conditioning circuit followed by the low-pass filter allowed a signal passing frequency range of 0.15–1600 Hz. Assuming ideal operational amplifier characteristics, the transmission gain is expressed as:

(10) $$\begin{array}{cc} G(s) & =-\frac{R_{f}\cdot \frac{1}{sC_{f}}}{R_{f}+\frac{1}{sC_{f}}}\cdot \frac{1}{R_{0}+\frac{1}{sC_{p}}}\cdot \frac{1}{\left(1+s\cdot R_{1}C_{1}\right)}\cdot \frac{1}{C_{p}}\\ \; & =\frac{sR_{f}}{\left(R_{0}R_{f}C_{p}C_{f}\cdot s^{2}+\left(R_{0}C_{p}+R_{f}C_{f}\right)\cdot s+1\right)}\cdot \frac{1}{\left(1+s\cdot R_{1}C_{1}\right)} \end{array}$$

**Figure 6:**
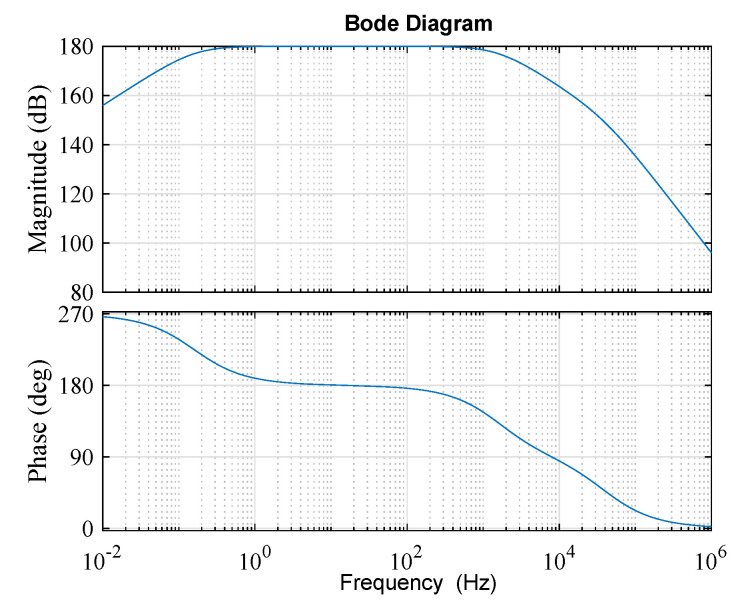
Bode diagram of the signal conditioning circuit.

The frequency response amplitude and phase are illustrated by the Bode diagram in Figure 6. The designed conditioning circuit could realize a sufficient amplification for the sensitive PVDF sensor and effectively remove the high frequency noise with a high signal-to-noise ratio. The signal could be processed in real time in a wide range of frequencies because of the negligible phase shift in this inverting amplifier circuit. The output voltage is calculated in Equation (11). In the denominator of the first factor, the rest of the terms are negligible because they are four orders of magnitude less than $C_{f}$. Therefore, the governing equation of a charge amplifier’s output voltage can be further reduced to:

(11) $$\begin{array}{cc} V_{O}=Q\cdot G\left(s\right) & =-\frac{Q}{\left(R_{0}C_{p}C_{f}\cdot s+\frac{R_{0}C_{p}}{R_{f}}+C_{f}+\frac{1}{sR_{f}}\right)}\cdot \frac{1}{\left(1+sR_{1}C_{1}\right)}\\ \; & ≃-\frac{Q}{C_{f}}\cdot \frac{1}{\left(1+sR_{1}C_{1}\right)} \end{array}$$

Furthermore, in the range of frequency concentrated on, $R_{1}C_{1}$ was much less than one. Therefore, this factor only reflected the feedback capacitance $C_{f}$.

(12) $$V_{o}≃-\frac{q}{C_{f}}$$

which eliminates the effects of both the piezo film and the connecting cable.

## 3. Numerical Simulation of Shear Strain Field

**Figure 7:**
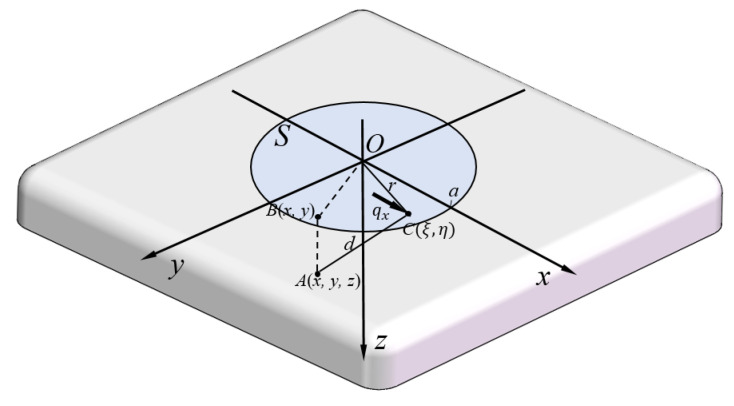
Tangential traction *q* acting over the loaded area *S*.

To demonstrate mathematically the shear strain distribution within the soft substrate, the contact mechanics of an elastic half-space loaded with a surface tangential traction was analyzed to simulate the shear strain numerically. A graphical representation is shown in Figure 7. Contact mechanics provides a theoretical basis for calculating the stresses and deformations in an elastic half-space under the action of surface traction. According to the theory of elasticity [31], small displacements of the particles of a deformed body are usually resolved into components *u*, *v*, and *w* parallel to the coordinate axes *x*, *y*, and *z*, respectively.

In this study, the stresses and deformations were produced in an elastic half-space, bounded by the plane surface z = 0, under the action of tangential tractions applied to the closed area *S* of the surface. Outside the loaded area, both normal and tangential tractions were zero. The two-dimensional surface tangential tractions $q_{x}\left(x,y\right)$ varied in both the *x* and *y* directions.

The half-space is shown in Figure 7. *C* is a general surface point of a concentrated force within the loaded area *S*, denoted at the position of (ξ,η,0). A(x,y,z) is a general point within the elastic body. The distance is:

(13) $$CA\equiv d={\left\{{\left(\xi -x\right)}^{2}+{\left(\eta -y\right)}^{2}+z^{2}\right\}}^{1/2}$$

According to our experimental conditions of no slip at the contact interface, it was important to investigate the formula for tangential traction $q_{x}\left(\xi ,\eta \right)$ over the loaded area *S*. The contact condition was set as a constant shear displacement of the surface within the circle. The shear deformation through adhesion was given by a tangential displacement parallel to the *x* axis to the surface of an elastic half-space. The tangential traction distribution $q_{x}\left(x,y\right)$ in the xy plane would not be uniform, but rather a radial pattern expressed by Equation (14),

(14) $$q_{x}\left(\xi ,\eta \right)=q_{0}{\left(1-r^{2}/a^{2}\right)}^{-1/2}$$

(ξ,η) is within the range of a circle of radius *a* [32]. This relation can be confirmed by deriving the surface displacement, which is a constant independent of *x* and *y*.

The deformed displacement can then be derived as:

(15) $$\begin{array}{cc} u= & \frac{1}{4\pi G}\int_{S}\int q_{x}\left(\xi ,\eta \right)\\ \; & \times \left\{\frac{1}{d}+\frac{1-2\nu }{d+z}+\frac{{\left(\xi -x\right)}^{2}}{d^{3}}-\frac{\left(1-2\nu \right){\left(\xi -x\right)}^{2}}{d{\left(d+z\right)}^{2}}\right\}d\xi d\eta \end{array}$$

where:

(16) ξ=rcosϕ,η=sinϕ

as shown in research on contact mechanics [32].

Since a simple shear deformation was applied in this experiment, only a one-dimensional shear strain distribution $\gamma_{xz}=\frac{\partial u}{\partial z}$, which can be calculated from Equation (15), was considered in the analysis. The physical parameters of the artificial skin substrate, Young’s modulus *E* and Poisson’s ratio ν, were taken to be $1.39\times 10^{5}$ Pa and 0.28, respectively.

According to the theory of elasticity, the equations were derived under the condition that the area of the contact patch was small in comparison with the areas of the bodies themselves. However, in contrast to the ideal condition, the boundary condition of the elastic half-space in our experimental circumstances was not restricted to the contact interface. The bottom edge was also restricted to maintain a displacement of zero.

**Figure 8:**
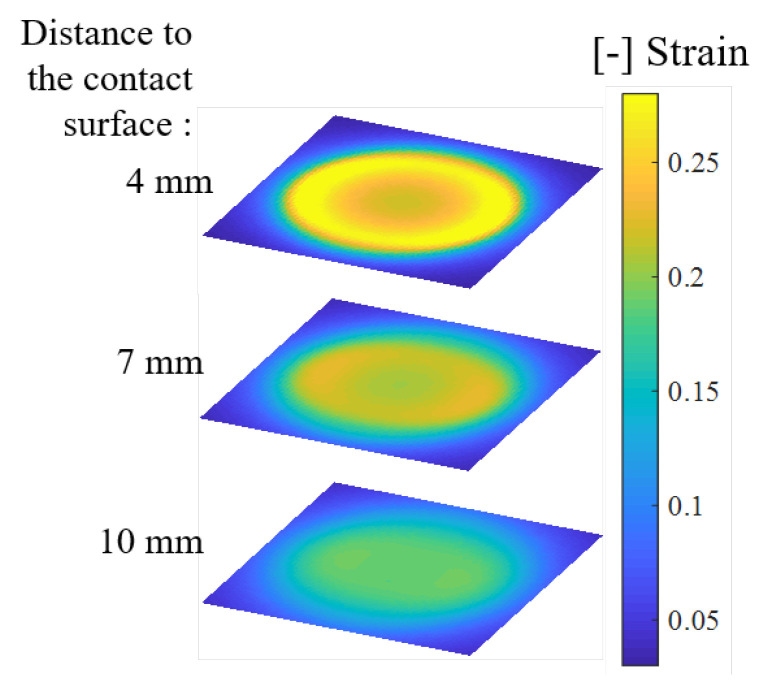
Simulated shear strain distribution under uni-directional shear tractions on circular regions in the stress direction.

By superimposing the effects of the two types of boundaries in calculating the strain component of the substrate, the shear strain distribution under the round contactor is plotted as shown in Figure 8.

## 4. Shear Strain Distribution Measurement

### 4.1. Experimental Design and Setup

**Figure 9:**
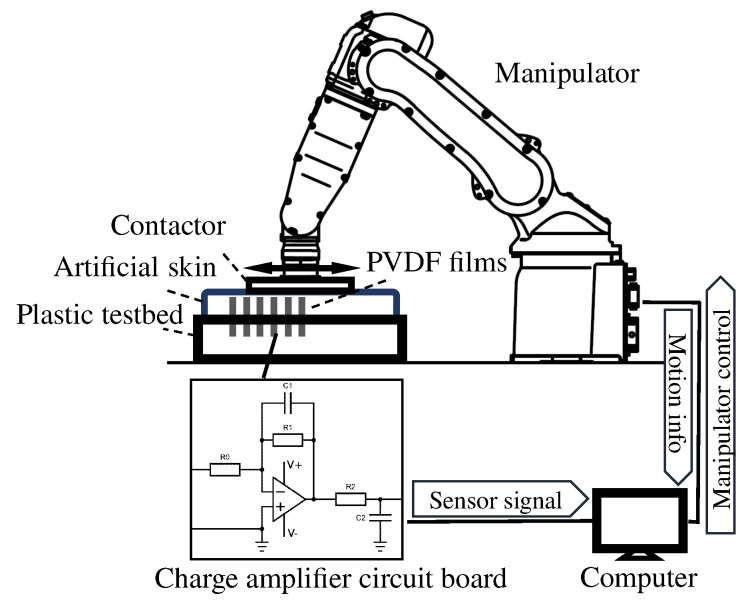
Experimental system.

The apparatus and experimental system are illustrated in Figure 9. In this research stage, a type of artificial skin (HITOHADA GEL, EXSEAL Co., Ltd., Mino, Japan) with an ASKER-C hardness of zero was utilized as the soft substrate material. It is a type of urethane resin that is capable of providing effective robustness during mechanical deformation and has the ability to adapt to changes in the environment for the experimental requirement. Artificial human skin is commonly employed in such experiments, considering ethical issues and experimental convenience. A plastic testbed with inlaid clamps matching the films’ thickness was manufactured as the PVDF film supporter using a three-dimensional (3D) printer. The electric wiring from the solder tabs for signal transmission was performed below the testbed.

The external stress was applied with a manipulator (MOTOMAN-MH5F, Yaskawa Electric Co., Ltd., Kitakyushu, Japan). The dynamic simple shear deformation was controlled within an amplitude range of 0 to 5 mm and a frequency range of 0 to 5 Hz. The range and frequency of motion were determined based on previous studies that measured the interactive force characteristics and deformation of skin surfaces in contact with a physical assistant robot. The variation of the relative positional relationship between the knee joint rotation center and the attached lower-leg part during knee joint flexion was detected in the range of 0 to 15 mm [33]. The frequency of the cuff offset during the usage of the physical assistant robot was collected up to 10 Hz and analyzed by Fourier transform [34]. Representative sustainable usage of the experimental apparatus was also considered.

**Figure 10:**
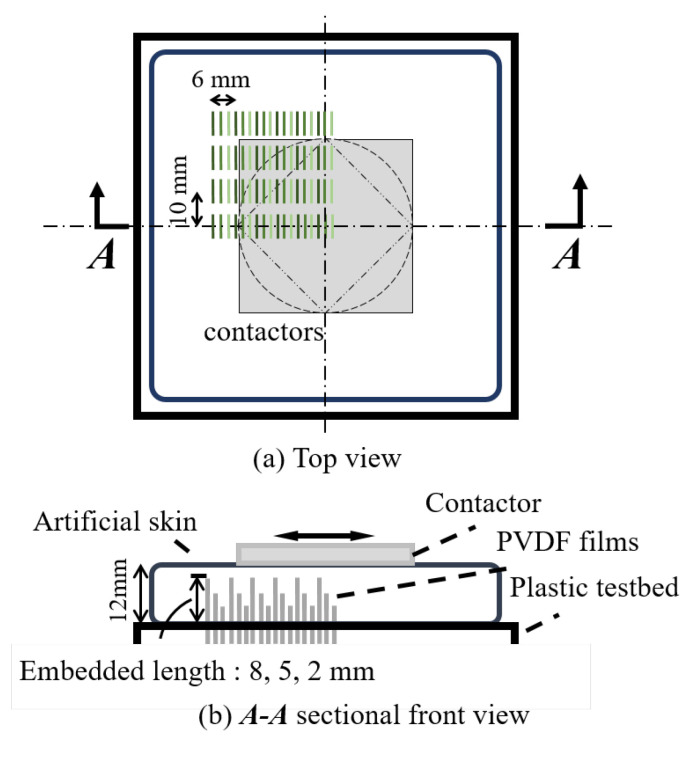
PVDF films’ arrangement.

The arrangement of PVDF elements within the artificial skin is schematically illustrated in Figure 10. The experiment was designed for central symmetric measurement with PVDF films arranged in one quadrant of the field to explain the whole range of the central symmetrical area.

In each sensing unit, three identical PVDF films were arranged with different embedded lengths of 8, 5, and 2 mm. The remaining part of the sensor was extended and fixed under the testbed. A 4 × 6 matrix of 24 units of 72 PVDF films was arranged with the units at intervals of 2 mm in the shear deformation direction and 10 mm in the lateral direction. Each PVDF sensing element was connected to an independent charge amplifier circuit in one trial of the experiment. Since there was no multiplexing and the number of circuits was limited to 12, in one trial, the 72 elements were divided into six trials measured under the same condition. The contactors were designed in three representative central symmetrical shapes of aluminum blocks: a square 50 mm × 50 mm in size, a circle 50 mm in diameter, and a square diamond 35 mm × 35 mm in size. All of these were in contact with the central position of the substrate surface. The artificial skin material was manufactured to a thickness of 12 mm. It was loaded in a sinusoidal pattern with an amplitude of 2.5 mm and a frequency of 2 Hz. It also should be noted that the contact interface had no slip.

### 4.2. Calibration

Before the measurement, the multi-sensor system needed to be calibrated to ensure the consistency of each PVDF element. In our case, the measurement value had no reference criterion for use in normal calibration, so the consistency was statistically standardized. The output of the signal should always be the same if the element is deformed by the same gesture. Every unit was tested under the same surface deformation according to the same pattern as described in Section 4.1. In every trial, it was confirmed that the tested unit was located at the center of the contactor. The amplitude of the wave form of the output charge generation from every PVDF film element was obtained and taken as the calibration factor. The mean values corresponding to the different embedded lengths were taken as the reference criteria. The ratio of the measured value to the criterion for each element was obtained and used to process the measurements obtained during the experiment.

### 4.3. Measurement Result

**Figure 11:**
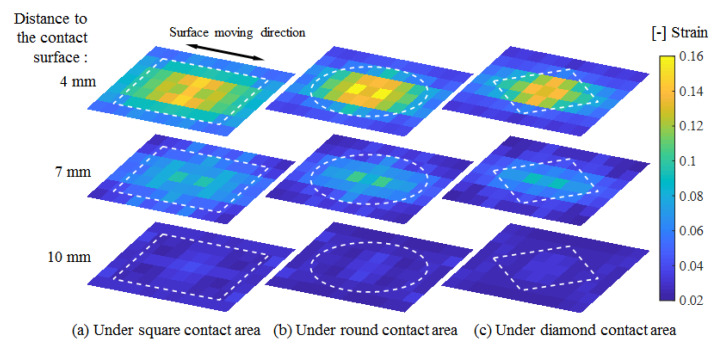
Distributions of shear strain amplitude detected from 24 units of PVDF films extended by the central symmetry under three contact shapes plotted under the same range scale.

The shear strain amplitude under sinusoidal loading measured from 24 units of PVDF films was used as the representative parameter for comparison among conditions. The distribution of shear strain from one of the quadrants was extended to the whole plane by central symmetry, as shown in Figure 11. In the depth direction, the distances to the surface corresponded to the different embedded lengths of the PVDF film. The white dotted lines represent the contact areas.

In this experiment, we obtained a shear strain field within an artificial skin in the range of 0.02 to 0.16. The distribution illustrated that high strain occurred close to the contact surface and decreased rapidly toward the bottom region. In addition, strain mainly clustered under the contact area, extended in the deformation direction, and diminished toward the border of the elastic body. Moreover, the decreasing tendency from the center to the border also existed over the range of the contact area, although the contact surface deformation was constant.

Among the three contact shapes, a wider distribution of high strain occurred under the larger area of the square contact. The distribution at the contact edge almost agreed with the contact shape, especially at the front edge relative to the motion direction. The central area exhibited a complex strain distribution and variation among the different contact conditions.

## 5. Discussion

The measurement results indicated that the internal shear strain field within an artificial skin could be visualized by the proposed sensor system. In the depth direction, large shear strain within the substrate material was mainly concentrated near the contact surface. These results were consistent with the generation of friction blisters and skin abrasions, which frequently occur in the epidermis and dermis of the skin. The shear strain distribution was found to be related to the force direction and differed for different contact shapes. Different strain concentrations at the edge region were detected. This sensor system had sufficient sensitivity to be able to distinguish among different contact shapes. This sensor system has the potential to contribute to the assessment of machinery safety in human-robot collisions. To contribute to the further development of the fundamental science through obtaining more information on stress and the stress-strain relation, the physical properties of biomaterial with complex structures, such as the elastic modulus, should be also measured.

The simulated shear strain distribution tendency was reflected well in the measurement results. The complex strain generation observed in the central area was consistent with the results obtained. A rapid decrease in the depth direction was also confirmed. Therefore, the sensing model established was confirmed to be an appropriate mathematical explanation of the sensing principle. This sensor system could serve as an effective and practical method for the measurement of the shear strain distribution in soft material.

**Figure 12:**
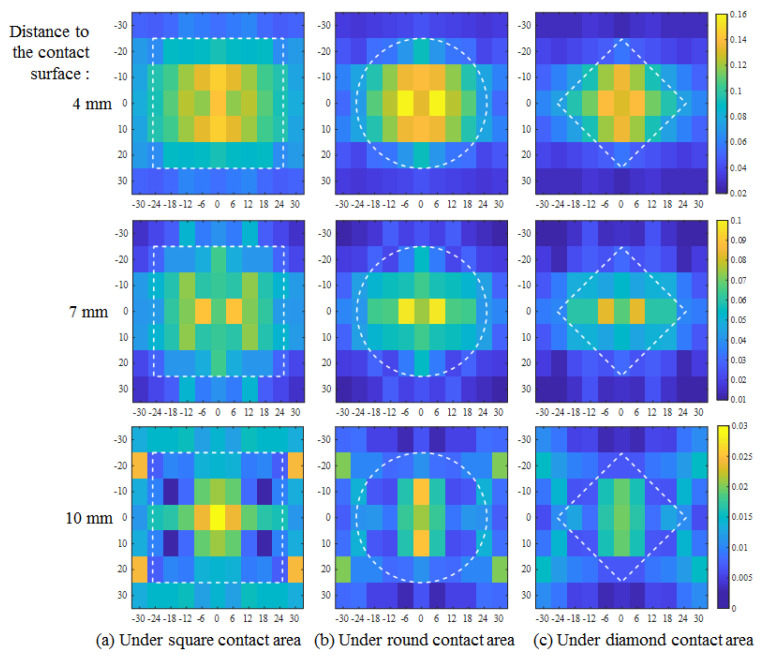
Distributions of shear strain amplitude detected from 24 units of PVDF films extended by central symmetry under three contact shapes plotted on the responding range scale.

In analyzing the measurements in detail, the results shown in Figure 11 are replotted on the responding range scale as shown in Figure 12. At each depth, although the distribution generally reflected a tendency to decrease from the center to the edge, as presumed, an abnormal discontinuity still existed. For example, in the plane 10 mm from the surface, an overly large value occurred at the front edge position. The calibration method therefore needed improvement, because some unknown installation error might occur with susceptible PVDF films. The deflection of the PVDF bending beam should be monitored using a camera or other distance sensor to achieve more precise calibration for more stable performance in different structures.

Additionally, for a wider range of application, miniaturization of the design should be considered. Although the substrate artificial skin had a great compression set and sufficient robustness for the experimental requirements, the life of the sensor system would depend on the robustness of the substrate, which was subject to deterioration.

## 6. Conclusions

In this study, a sensor system that could measure the internal shear strain distribution of soft material using built-in flexible PVDF films was developed. This novel sensor system took an implantable structure and created a new type of measurement method to overcome the limitations of conventional interactive force sensors in their ability to measure and estimate strain distributions within soft materials. A sensing model was mathematically established to explain the sensing principle. Arranging the sensing element of the PVDF well made it possible to conduct an experiment in three-dimensional measurement of the shear strain distribution within an artificial skin. A consistency calibration method that was applicable to multi-sensor systems was employed. The proposed sensor system could visualize the shear strain field within an artificial skin and was sensitive to different contact conditions. A numerical simulation based on contact mechanics was conducted to verify the measurement results. The results confirmed that the proposed sensor system, which used built-in PVDF films, could be used to measure the distribution of shear strain within a soft material. This study could significantly contribute to the analysis of the skin damage and the skin contact safety assessment because the proposed sensor system could quantitatively visualize the three-dimensional shear strain distribution and could measure the stress concentration state under different contact conditions of an artificial human skin. The improvement of the accuracy, which will be achieved by improving the calibration process, is essential for future practical application of this sensor system.

## 7. Patents

A patent has been applied for the research results (patent application number: 2019-179219). 

## References

[1] Hassan P., Verma G., Ganguly R. (2011). Soft materials-properties and applications. Functional Materials: Preparation, Processing and Applications.

[2] Mao X., Yamada Y., Akiyama Y., Okamoto S. (2017). Characteristics of Dummy Skin Contact Mechanics During Developing Process of Skin Abrasion Trauma. Tribol. Lett..

[3] Bashir S.J., Chew A.L. (2016). Mechanical injury to the skin. Rook’s Textbook of Dermatology.

[4] Sanders J.E., Goldstein B.S., Leotta D.F. (1995). Skin response to mechanical stress: Adaptation rather than breakdown-a review of the literature. J. Rehabil. Res. Dev..

[5] Ito M., Pramudita J.A., Watanabe R., Shimizu Y., Tanabe Y. (2017). Investigation of skin laceration threshold under a specific condition: Blade penetration test on porcine skin. J. Mech. Med. Biol..

[6] Akiyama Y., Okamoto S., Yamada Y., Ishiguro K. (2015). Measurement of contact behavior including slippage of cuff when using wearable physical assistant robot. IEEE Trans. Neural Syst. Rehabil. Eng..

[7] Mao X., Yamada Y., Akiyama Y., Okamoto S., Yoshida K. (2017). Safety verification method for preventing friction blisters during utilization of physical assistant robots. Adv. Robot..

[8] Naylor P. (1955). Experimental friction blisters. Br. J. Dermatol..

[9] Naylor P. (1955). The skin surface and friction. Br. J. Dermatol..

[10] Jacobs T., Virk G.S. ISO 13482-The new safety standard for personal care robots. Proceedings of the ISR/Robotik 2014 41st International Symposium on Robotics.

[11] Grey J.E., Harding K.G., Enoch S. (2006). Pressure ulcers. BMJ.

[12] Akiyama Y., Yamada Y., Okamoto S. (2015). Interaction forces beneath cuffs of physical assistant robots and their motion-based estimation. Adv. Robot..

[13] Ozioko O., Karipoth P., Hersh M., Dahiya R. (2020). Wearable Assistive Tactile Communication Interface based on Integrated Touch Sensors and Actuators. IEEE Trans. Neural Syst. Rehabil. Eng..

[14] Dahiya R.S., Metta G., Valle M., Sandini G. (2009). Tactile sensing—from humans to humanoids. IEEE Trans. Robot..

[15] Yao K., Kaboli M., Cheng G. Tactile-based object center of mass exploration and discrimination. Proceedings of the 2017 IEEE-RAS 17th International Conference on Humanoid Robotics (Humanoids).

[16] Kaboli M., Feng D., Cheng G. (2018). Active tactile transfer learning for object discrimination in an unstructured environment using multimodal robotic skin. Int. J. Humanoid Robot..

[17] Feng D., Kaboli M., Cheng G. (2018). Active prior tactile knowledge transfer for learning tactual properties of new objects. Sensors.

[18] Lenzi T., Vitiello N., De Rossi S.M.M., Persichetti A., Giovacchini F., Roccella S., Vecchi F., Carrozza M.C. (2011). Measuring human–robot interaction on wearable robots: A distributed approach. Mechatronics.

[19] Noda K., Hoshino K., Matsumoto K., Shimoyama I. (2006). A shear stress sensor for tactile sensing with the piezoresistive cantilever standing in elastic material. Sens. Actuators A Phys..

[20] Shih B., Shah D., Li J., Thuruthel T.G., Park Y.L., Iida F., Bao Z., Kramer-Bottiglio R., Tolley M.T. (2020). Electronic skins and machine learning for intelligent soft robots. Sci. Robot..

[21] Yogeswaran N., Dang W., Navaraj W.T., Shakthivel D., Khan S., Polat E.O., Gupta S., Heidari H., Kaboli M., Lorenzelli L. (2015). New materials and advances in making electronic skin for interactive robots. Adv. Robot..

[22] Luo M., Liu D., Luo H. (2016). Real-time deflection monitoring for milling of a thin-walled workpiece by using PVDF thin-film sensors with a cantilevered beam as a case study. Sensors.

[23] Ma C.C., Chuang K.C., Pan S.Y. (2011). Polyvinylidene fluoride film sensors in collocated feedback structural control: Application for suppressing impact-induced disturbances. IEEE Trans. Ultrason. Ferroelectr. Freq. Control.

[24] Shirinov A.V., Schomburg W.K. (2008). Pressure sensor from a PVDF film. Sens. Actuators A Phys..

[25] Yi J., Liang H. (2008). A PVDF-based deformation and motion sensor: Modeling and experiments. IEEE Sens. J..

[26] Yi J. (2008). A piezo-sensor-based “smart tire” system for mobile robots and vehicles. IEEE/ASME Trans. Mechatron..

[27] Li F., Akiyama Y., Wan X., Yamada Y., Okamoto S. Shear Stress Sensor for Soft Material with Built-In Piezoelectric Polymer Films. Proceedings of the 2019 IEEE 8th Global Conference on Consumer Electronics (GCCE).

[28] Specialties M. Piezo Film Sensors Technical Manual. https://www.sparkfun.com/datasheets/Sensors/Flex/MSI-techman.pdf.

[29] Sirohi J., Chopra I. (2000). Fundamental understanding of piezoelectric strain sensors. J. Intell. Mater. Syst. Struct..

[30] Beer F.P., Johnston R., Dewolf J., Mazurek D. (1981). Mechanics of Materials.

[31] Timošenko S.P., Goodier J.N. (1951). Theory of Elasticity.

[32] Johnson K.L., Johnson K.L. (1987). Contact Mechanics.

[33] Kengo Y., Yoji Y. (2014). Development of Specialized Dummy Coating for Viscoelasticity of Skin for Dummy for Safety Tests. Graduation Thesis.

[34] Akiyama Y., Yamada Y., Ito K., Oda S., Okamoto S., Hara S. Test method for contact safety assessment of a wearable robot-analysis of load caused by a misalignment of the knee joint. Proceedings of the 2012 IEEE RO-MAN: The 21st IEEE International Symposium on Robot and Human Interactive Communication.

